# The Piper Calls the Tune

**Published:** 1921-02-05

**Authors:** 


*To the Editor of* The Hospital.

### The Piper Calls the Tune.

*To the Editor of* The Hospital.

Sir,—The system of levying workpeople with the object of providing funds for the upkeep of the hospitals may be a necessary one, and the noble work done in the hospitals of this country merits all praise and is deserving of continuous support. But, like many another laudable project, the idea of a weekly levy stands in danger of being smothered by imitators; although Leicester and other towns may have solved temporarily their difficulties by thus transferring their burdens to the shoulders of the proletariat in a series of easy payments.

Take, for example, an artisan at a moderately remunerated calling and accepting a county-area rate of wages. One finds that he must, as a rule, belong to a trade union, whose permission to start work is coupled with a demand for a permanent weekly levy of a few shillings towards its working expenses. The Government requires another levy to provide for the nation's health, and, still later, a further permanent levy to meet the contingency of unemployment.

Another levy the worker agrees to support is in connection with a hospital or convalescent institution more

**THE EDITOR'S LETTER-BOX,—(*continued*).**

particularly connected with his individual trade. It has happened, also, upon a man further agreeing to a levy of an extra sixpence, say, for the National Institution of the Blind, or some equally meritorious cause, he is met at his workplace by the enterprising representative of a business house, which sees in the notion of a weekly levy a ground well prepared for the disposal of their wares on the instalment plan.

The thought arises that more energetic representations should be made to the employers of labour. By the small permanent levy to which the employee agrees upon his present and future earnings, absolute freedom from liability is accorded the employer. This may be in respect to workers who contract, as a result of the occupation, a disease that can be satisfactorily treated only in hospital.

The necessary permission to approach his workpeople with the view of a levy can scarcely be included as the redemption of a financial obligation on the part of the employer. Hamelin.

